# Environment-Friendly, Self-Sensing Concrete Blended with Byproduct Wastes

**DOI:** 10.3390/s20071925

**Published:** 2020-03-30

**Authors:** Marat Konkanov, Talal Salem, Pengcheng Jiao, Rimma Niyazbekova, Nizar Lajnef

**Affiliations:** 1 Technical Faculty, Saken Seifullin Kazakh AgroTechnical University, Nur-Sultan 010011, Kazakhstan; konkanov@msu.edu (M.K.); rimma.n60@mail.ru (R.N.); 2 Department of Civil and Environmental Engineering, Michigan State University, East Lansing, MI 48824, USA; salemtal@msu.edu (T.S.); lajnefni@msu.edu (N.L.); 3 Ocean College, Zhejiang University, Zhoushan 3216021, China

**Keywords:** environment-friendly, self-sensing concrete, compressive strength, electrical conductivity, byproduct wastes

## Abstract

Smart structures have attracted significant research attention in the last decade, mainly due to the capabilities of advanced concrete in electrical resistance-enabled self-sensing. In this study, we present a type of environment-friendly, self-sensing concrete enabled by electrical resistance. Environment-friendly, self-sensing concrete was casted with the additions of byproduct wastes (i.e., coal fly ash (FA), blast furnace slag (BOF) and red mud (RM)) at various volume fractions and cured using the conditions of 3, 7 and 28 days. The self-sensing concrete samples were experimentally tested to investigate the effects of the byproduct wastes on the mechanical and electrical properties (i.e., compressive strength and electrical resistance). In the end, parametric studies were experimentally conducted to investigate the influences of the byproduct wastes on the mechanical and electrical properties of the reported environment-friendly, self-sensing concrete.

## 1. Introduction 

Approximately over 30 million tons of concrete are consumed worldwide each year, making concrete the second most used resource after water [1]. The desired balance between performance (e.g., strength, durability, etc.) and cost offered by concrete leads it to be one of the most extensively used materials in constructions [2,3]. Therefore, partial replacement of Portland cement with supplementary cementitious materials has become a widespread strategy that positively affects the sustainability of concrete productions [4]. Among its counterparts in the field, blended cement has been preforming promising results in terms of the reduction of the carbon footprint, energy content and the durability of the concrete-based infrastructure [5,6,7]. 

Studies in the literature have demonstrated that properties of Portland cement such as electrical conductivity can be affected applying certain fillers to its matrix [8,9,10,11]. Electrical conductivity of concrete has attracted research attentions during the past decades [12,13,14,15,16,17,18]. Electrical conductivity can be increased if certain quantities of conductive materials are applied to ordinary concrete, which leads to a concrete with advanced mechanical and electrical properties. However, the high cost of these nanofillers is a main issue that has limited their applications. To address the issue of cost, studies have reported achieving low electrical resistivity using by-product wastes as fillers [19]. Waste materials are abundant in alumina, silica and iron oxides, which make the latter good candidates for conductive additives in concrete and mortars [20,21,22]. More recently, research attentions have shifted to investigating the electrical resistance of concrete for self-sensing. Electrical conductivity is one of the main factors that affect the self-sensing ability of the concrete. Typically, fibers are more common additives for obtaining good electrical conductivity as they may form conductive networks [23,24,25,26,27,28,29,30]. Thanks to its electrical resistance, self-sensing concrete has been reported in many fields. The electrical conductivity of concrete has a large influence on self-heating, self-healing and self-diagnosing properties of concrete [31,32,33,34,35,36]. It is necessary to improve the electrical properties (e.g., conductivity) of concrete since it assists in addressing the key industrial needs related to, for example, vibration control, SHM, building safety and security and rising costs of managing vast aging infrastructures [37,38]. However, the majority of the existing studies have investigated the electrical resistance of self-sensing concrete using the complex plasticizers and admixtures. As a consequence, it is of research and practical interest to obtain an advanced understanding on the mechanical and electrical properties of environment-friendly, self-sensing concrete.

Here, we study a type of environment-friendly, self-sensing concrete enabled by electrical resistance. The reported concrete is self-sensing by using the fractional change in resistivity (FCR) to monitor and indicate the status of the concrete. We first fabricated the self-sensing concrete with the additions of byproduct wastes, including coal fly ash, blast furnace slag and red mud. Three curing conditions of 3, 7 and 28 days were particularly investigated. Cement mortars prepared with Portland cement type I were examined with the byproduct wastes at different volume fractions. The compressive strength and electrical resistance of the environment-friendly, self-sensing concrete samples were experimentally investigated with respect to the byproduct wastes. To accurately reveal the status of the concrete, it is necessary to investigate the relationships between the byproduct wastes and the performance. Therefore, we experimentally calibrated the mechanical and electrical characteristics of the self-sensing concrete. Finally, parametric studies were experimentally conducted to investigate the influences of the byproduct wastes on the mechanical and electrical properties of the self-sensing concrete. 

## 2. Materials and Preparation of the Environment-Friendly, Self-Sensing Concrete Samples 

In this study, different dosages of the blended additives, i.e., coal fly ash type F (FA), blast furnace slag (BOF) and red mud (RM), were used to partially replace the Portland cement in the environment-friendly, self-sensing concrete samples. The chemical compositions composition of the byproduct wastes (i.e., coal fly ash, blast furnace slag and red mud) were obtained via x-ray fluorescent spectrometry (XRF); the loss on ignition results are presented in Table 1. The coal fly ash and blast furnace slag had a weight ratio of SiO_2_/Al_2_O_3_ equal to 1.53, 4 and a weight ratio of SiO_2_/CaO of 3.3 and 0.35, respectively (i.e., based on the values given in italic in Table 1). In the case of red mud, the weight ratio of silica to alumina oxide and silica to calcium oxide were found to be 0.46 and 0.50, respectively.

**Figure 1:**
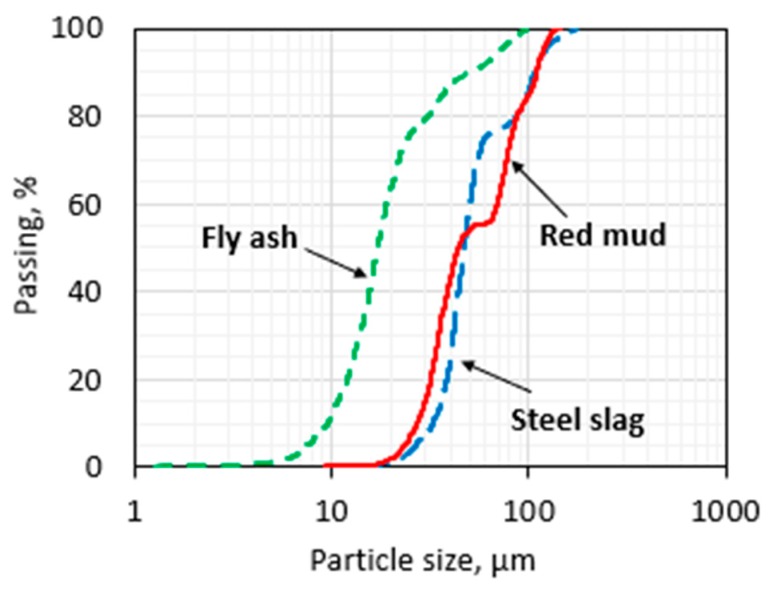
Particle size distribution.

The particle size distribution of the by-product wastes and Portland cement type I was measured via a 3071A Analyzer; the observations are presented in Figure 1. The median particle sizes for the coal fly ash, blast furnace slag and red mud was 18, 49 and 43 μm, respectively. In general, the fineness quality (specific surface area) of end-product depends on the grinding approach (e.g., ball milling, cryogenic grinding). However, the energy consumption was the main parameter that limited our particle size. Procedures and conditions for grinding both RM and BOF were same; however it can be seen in Figure 1 that the median particle size of RM is smaller than that of the steel slag, which may be explained by the fact that steel slag particles form under the influence of high temperature that is affected by the hardness of this material. It is worth mentioning that coal fly ash was used as obtained. 

The weight ratios of the solid raw materials used to produce the environment-friendly, self-sensing concrete were 5%, 10%, 15%, 20% and 25% of the total weight of Portland cement type I, as shown Table 2. A classic quart tilt-head stand mixer was used to mix all the materials: cement, waste products, silica sand and water. These ratios were devised to provide chemistry amenable adequate and desired electrical conductivity. The supplementary cementing materials were milled with ordinary Portland cement (OPC) for 8 h using a ball mill with a speed of 65 rpm and materials-to-balls ratio of 43%. The water/cement ratio of mortar mixtures was adjusted to produce a fresh mix flow of 110 ± 5%, based on the suggestion in the Standard Test Method for Flow of Hydraulic Cement Mortar given in ASTM C1437 [39]. The resulting water-to-cement ratios ranged from 0.50 to 0.56.

**Figure 2:**
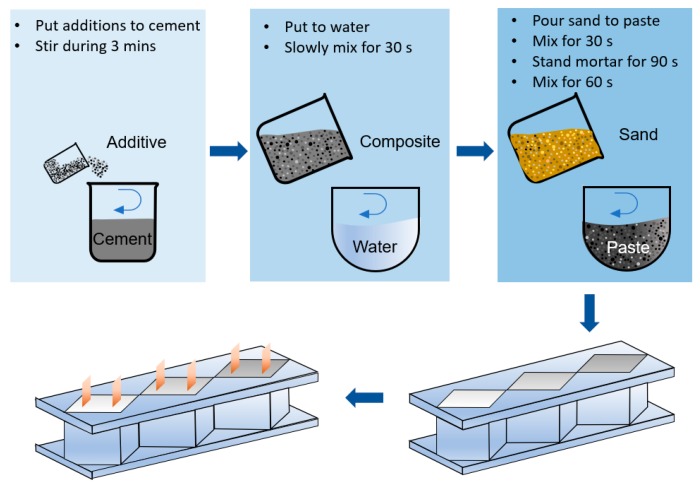
Mortar preparation process.

Production of the environment-friendly, self-sensing concrete samples was completed by following the steps shown in Figure 2, including (i) specified dosage of each of the waste materials was added to Portland cement and the mix was stirred for 3 min at low speed; (ii) water was added and the cement paste was stirred for 30 s; (iii) silica sand was gradually added with a 2.75 sand/cement ratio for all the sixteen specimens and the mortar was left to stand for 90 s; and finally (iv) the whole mortar was then mixed at medium speed for 60 s.

Specimens were casted in 50 mm cubic molds following ASTM C109 [40]; and two inner copper electrodes (25 × 50 × 0.2 mm) were placed at distance of 10 mm from the edges of each cube. A copper foil with 0.07 mm thick was attached by conductive silver paint to work as an external electrode. The specimens were shaken on a vibrating table to reduce air bubbles and ensure compaction. After 24 h, the specimens were demolded and placed into a curing room at 20 °C and relative humidity of 95%. Fly ash was obtained from Louisville Gas and Electric power plant in Louisville, Kentucky with median particle size of 18 μm and pH of 12.87 (bulk density 1260 kg/m^3^). The blast furnace slag had a bulk density of 2027 kg/m^3^ and was supplied by Phoenix-Services. The red mud had a specific gravity of 1.9 and was collected from Pavlodar alumina plant in Pavlodar, Kazakhstan that has a 1.5-million ton annual production of alumina [41].

## 3. Experimental Setup and Testing 

**Figure 3:**
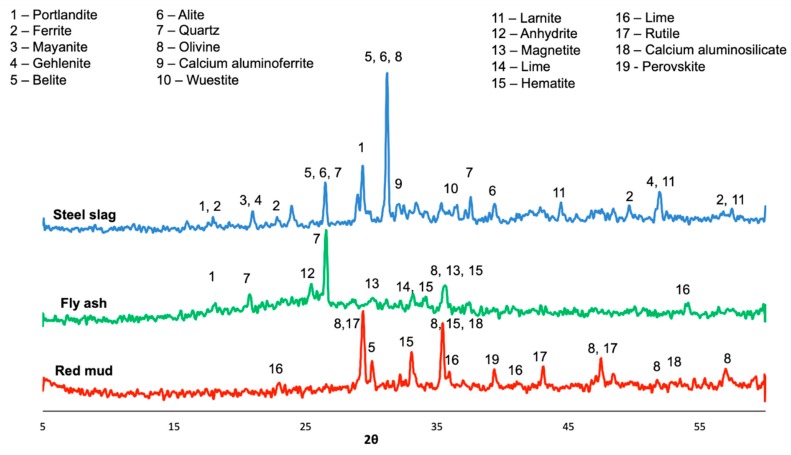
x-ray diffraction (XRD) results for red mud, fly ash and steel slag.

In the experiments, we first evaluated the microstructural characteristics of the environment-friendly, self-sensing concrete using x-ray diffraction (XRD) analysis and pH approaches. The performance of the self-sensing concrete was evaluated with respect to the electrical resistivity, setting time and compressive strength. Ordinary Portland cement (OPC) was used as a reference for comparing electrical conductivity. The mineralogy of the developed environment-friendly, self-sensing concrete was assessed using the x-ray diffraction (XRD) technique. A Bruker D8 x-ray diffractometer equipped with Cu x-ray radiation operating at 40 kV and 30 mA was used for performance of the XRD tests; the covering a reflection angle range 2θ was between 5–60°. A continuous PSD fast was adopted in the scan mode and the counting time for each step was set to be 0.2 s. pH was used to measure the 1% aqueous solution of the solid material using Fisher Scientific Accumet AB15 instrument. In this test, 1 g of the concrete powder was dissolved in 100 g of distilled water. The solution was placed on a shaker for 30 min at 200 rpm. The solution was left to settle for 30 min and then the pH value was measured. It can be seen from Figure 3 that 

BOF contained belite and alite phases in crystal form, and ferrite, mayanite and wuestite in amorphous form. The main components of FA were quartz, lime, magnetite and hematite, which were in the amorphous form as well. RM was in the amorphous phases of hematite, lime, rutile and aluminosilicate.

The compressive strength of 50-mm cubic mortar specimens was measured based on ASTM C109 at 3, 7 and 28 days of curing [42] using the FORNEY Compression machine Three specimens were tested and the average value of the compressive strength was recorded. The initial and final setting times of the cement pastes were measured based on ASTM C191 using the Vicat needle apparatus [43]. The amount of water mixed with cement was measured for the setting time [44]. Electrical resistance measurements were carried out using a BK Precision 4071A signal generator, Tektronix TDS 1002 two channel oscilloscope (with 3% accuracy) and Radio Shack digital multimeter (with 1.2% accuracy) with a voltage of 5 V and in the frequency range of 0.1–100 kHz. A frequency level of 10 kHz was chosen as the reference, since this was the effective range of the multimeter. 

**Figure 4:**
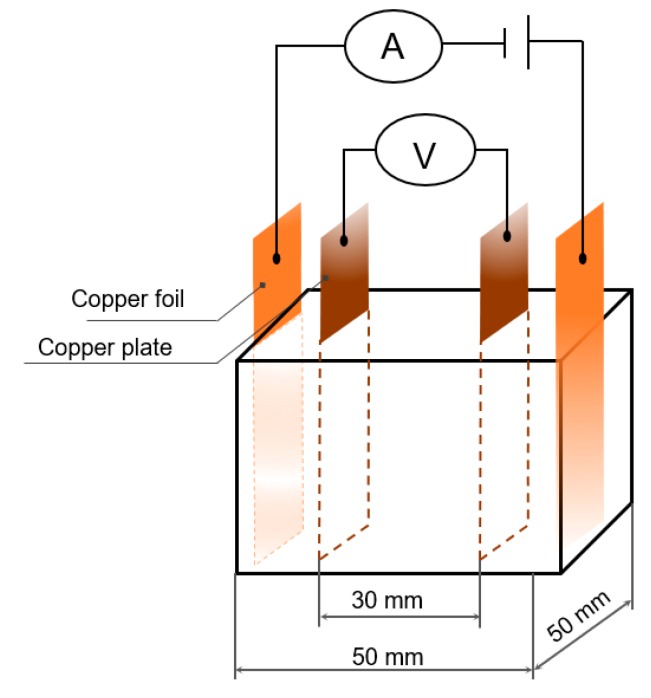
Sample and electrodes setup.

**Figure 5:**
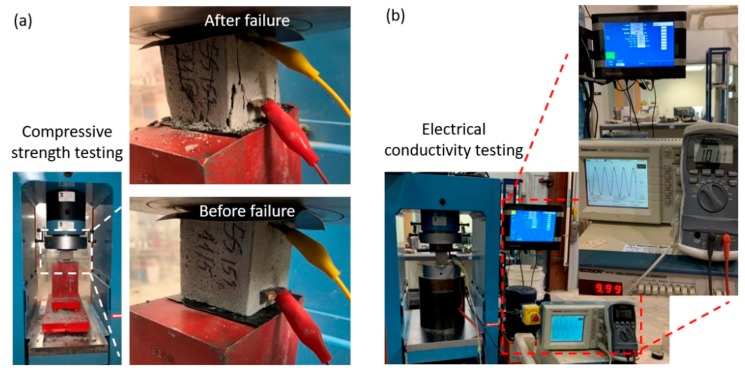
(**a**) Compressive strength test and (**b**) measuring electrical resistivity test.

Two embedded copper electrodes were used to assess the voltage parameters; AC current flows was measured between the two external foil electrodes. Due to contact resistance issues associated with the two-probe approach, a four-probe approach was adopted to measure the electrical resistance of the concrete samples, as shown in Figure 4 [45]. Figure 5 presents experimental setup and results of the mechanical and electrical testing. Figure 5a shows the mechanical testing and Figure 5b displays the electrical testing. 

The electrical resistivity of the mortar specimens, *ρ*, can be calculated as:

(1) $$\rho =\frac{V\cdot A}{I\cdot L},$$

where *V*, *I*, *A* and *L* are the voltage, current readings, the cross-sectional area and distance between the inner electrodes, respectively.

## 4. Experimental Results of the Compressive Strength and Electrical Resistivity

Table 3 presents the setting time and pH results of the waste materials. It can be seen that increasing the fly ash and steel slag dosage led to increasing initial and final setting times. However, red mud tended to have the inverse effect, i.e., increasing red mud reduced the setting time.

**Figure 6:**
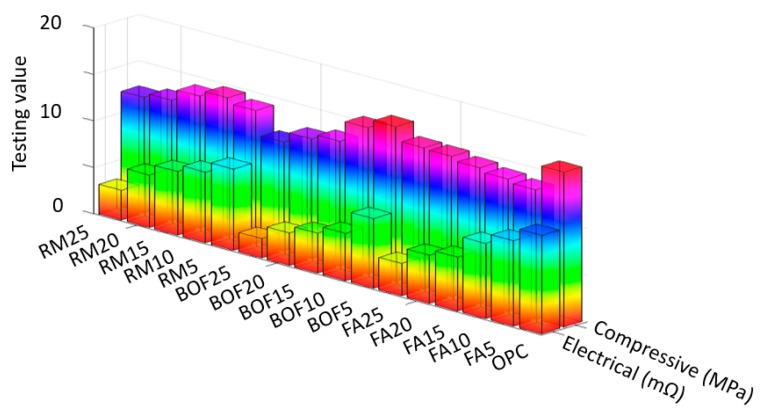
Compressive strength and electrical resistance results for the environment-friendly, self-sensing concrete cured at the age of 3 days.

**Figure 7:**
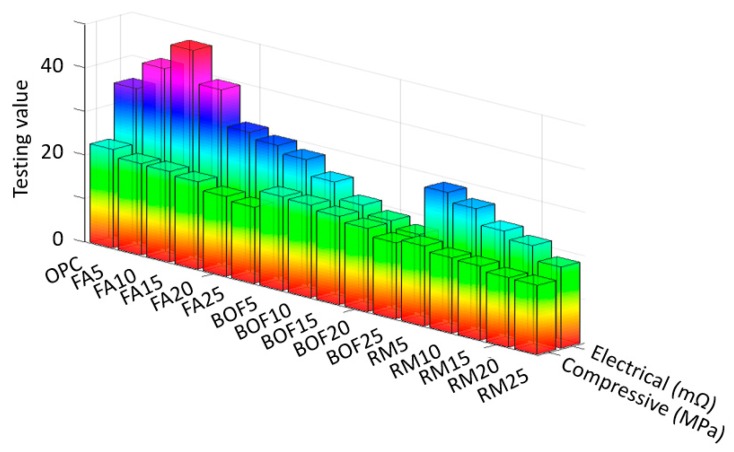
Compressive strength and electrical resistance results for the environment-friendly, self-sensing concrete cured at the age of 7 days.

**Figure 8:**
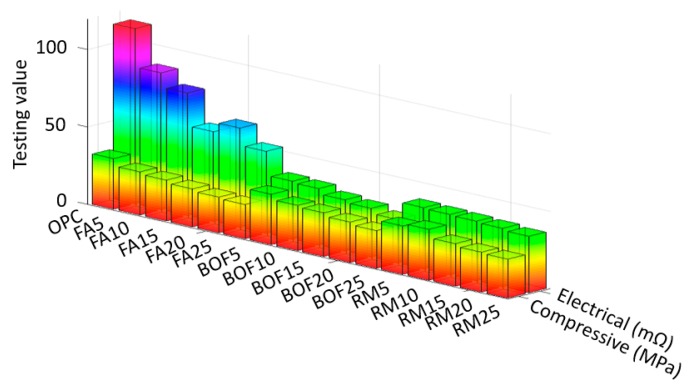
Compressive strength and electrical resistance results for the environment-friendly, self-sensing concrete cured at the age of 28 days.

Figure 6, Figure 7 and Figure 8 present, respectively, the experimental results of the compressive strength and electrical resistivity for each used waste material at the curing age of 3, 7 and 28 days (raw data are provided in Appendix A). To avoid measurement errors in the experiments, we repeated the same experiments three times; the presented raw testing data (i.e., the testing data in Table A1, Table A2 and Table A3 in the revised manuscript) are the mean values. Note that Figure 6, Figure 7 and Figure 8 aim to present the overall variation trends of the compressive strength and electrical resistance. To compare the exact testing values, however, it is necessary to use the raw testing data presented in Table A1, Table A2 and Table A3. According to the mechanical and electrical variation patterns, increasing the dosage of waste materials led to a decrease of compressive strength for all the specimens at early and late ages. In particular, the late age of 28 days in Figure 8—both the replacing 5% of Portland cement with BOF and 10% of Portland cement with RM—are obtained with similar results of OPC with 0.21% and 0.88% differences. This observed decrease in compressive strength may be due to the presence of crystalline tricalcium silicate and dicalcium silicate phases in the BOF as shown in Figure 3. In addition, the relatively high content of magnesium oxide in the BOF may have a negative effect on the final hardening of cement mortar [46]. In the case of fly ash, the compressive strength was improved by increasing the dosage of fly ash at the early age of 3 days. In particular, replacing 25% of Portland cement with fly ash gave the highest compressive strength compared with results of this additive at other ages. This strength development may be due to presence of aluminum oxide which participates in forming calcium aluminum hydrate [47]. The compressive strength of fly ash at late ages (i.e., 7 and 28 days), however, shows opposite trends compared with the one at the early age of 3 days. This may be explained by the low quantity of the calcium oxide, which limits the production of the C-S-H gel during hydration [48]. These interesting trends were observed when red mud was used as the additive material, which may be due to the presence of the calcium oxide; iron oxide; aluminum oxide and titanium oxide in the amorphous phase. Those additions showed positive influence on increasing the compressive strength of the cement mortars [49,50].

The electrical resistivity of the mortars was measured at the early and late ages without applying any mechanical load. Note that the results in Figure 6 are rotated by 180° as compared with the results in Figure 7 and Figure 8, because the electrical values were too small to be properly shown in the same order. Specimens were not completely dried (i.e., continuously cured until the test), and therefore, excess water content may explain the increasing trends of electrical resistivity with time due to the presence of ions in pore water of cement mortar [10,51,52]. All the tested specimens in Figure 6, Figure 7 and Figure 8 showed little electrical resistivity (including the OPC); however, BOF and RM showed the lowest values for resistivity, which is possible due to the electrolytic and electronic types of the conductions, as discussed in Han [11]. Introducing of the steel slag and red mud was likely to decrease the electrical resistivity of the OPC mortar by three times at the end of curing time, which may be due to the relatively high content of the metal oxides (i.e., Fe_2_O_3_) found in these byproducts, as shown in Table 1. On the third day, the electrical resistivity was decreased when the fly ash content was increased, which may be because the samples contained iron oxide.

## 5. Self-Sensing Property

In this study, the maximum fractional change in resistivity (FCR) is calculated as

(2) $$\mathrm{FCR}=\frac{\rho_{i}-\rho_{0}}{\rho_{0}},$$

where, $\rho_{0}$ and $\rho_{i}$ are readings of electrical resistivity measured at initial moment and during loaded state, respectively. 

**Figure 9:**
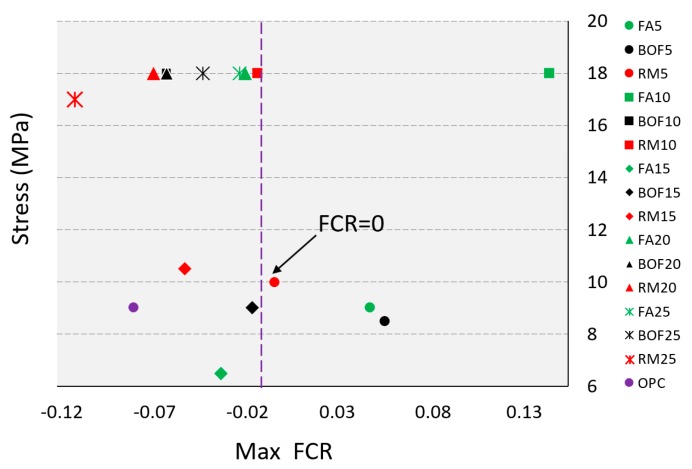
Maximum of fractional change of resistivity (FCR) during loading.

Figure 9 indicates the results of the maximum FCR, which are obtained under the axial loading of 10 and 18 MPa (i.e., which is approximately 45%–60% of the failure load for the self-sensing concrete). The matrix of the cement-based sensor was compacted under compressive loading, and therefore, the distance between the electrons in the cement matrix was reduced, which caused the decreasing of the electrical resistivity [15]. Thus, the FCR of specimens were observed with negative values for the compressive loading [53]. The specimens with high ranges of replacement materials were the most effective with self-sensing ability—and more interestingly—the specimens with 5% of additives did not obtain the necessary changes of electrical resistivity. However, it is worthwhile to point out that the accuracy of the maximum FCR can be critically affected when the concrete is failed under axial compression, and therefore, the self-sensing capability of the concrete is valid for up to 75% failure degree. Table 4 presents the stress sensitivity coefficient results for the cement mortars at the age of 28 days. 

Next, the stress sensitivity coefficient of the mortar was calculated as

(3) $$\mathrm{Stress}\;\mathrm{sensitivity}\;\mathrm{coefficient}=\frac{\max\left|\mathrm{FCR}\right|}{\delta }$$

where, FCR and δ are the maximum value of fractional change in the electrical resistivity and certain stress value during loaded state, respectively.

**Figure 10:**
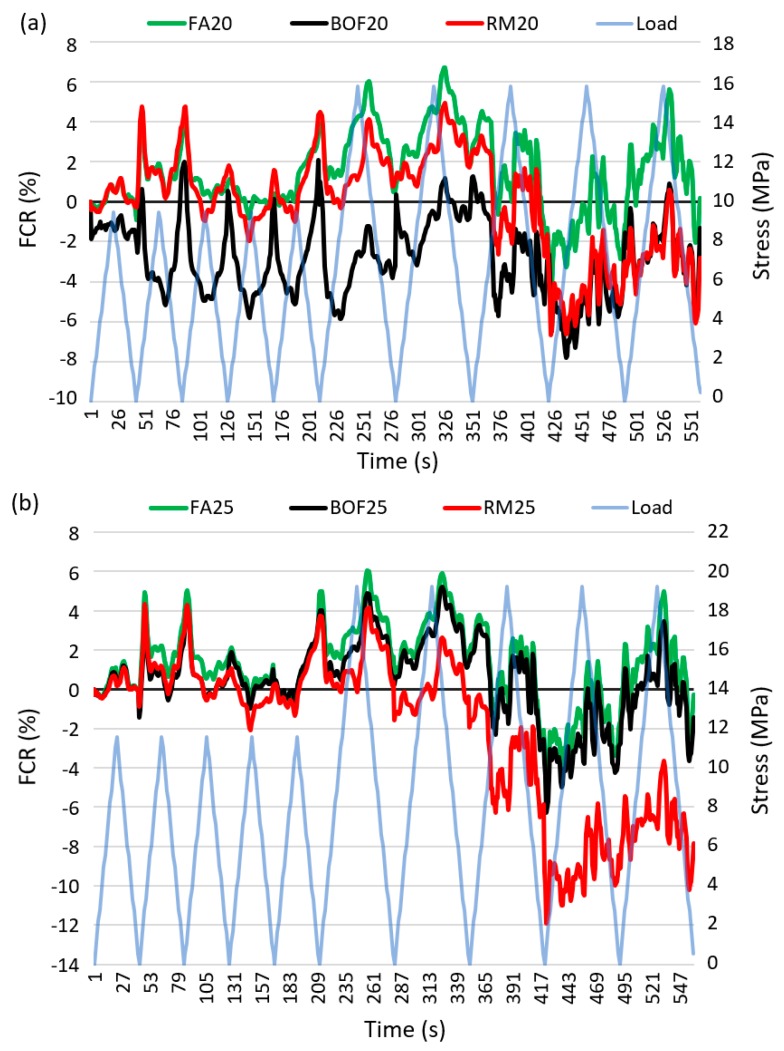
Fractional change of resistivity (FCR) under cycle loadings: (**a**) with 20% of byproduct waste (**b**) with 25% of byproduct waste.

Figure 10 shows the FCR of the mortar cement samples subjected to the cyclic loading of less than 18 MPa (i.e., approximately 45%–75% of compressive strength in Table A3). Figure 10a,b present FCR with 20% and 25% of byproduct wastes, respectively. The 20% and 25% replacement ratios were particularly used to investigate the patterns of FCR, since lower FCR values (see Figure 9) were obtained with 20% and 25% additions (i.e., FCR is expected to be more stable and reliable with those replacement ratios). Of all the tested specimens, FCR was critically affected by the used additions. 

Figure 10a presents the patterns of FCR for FA20, BOF20 and RM20. It can be seen that the FCR curves started fluctuating after 50 s which were somehow reduced after the loading time of 80 s. The variations were critical during 100–200 s with the overall increasing pattern and then started showing the opposite trends since approximately 440 s. This might be due to the significant irreversible damages caused in the specimen structure, and therefore, the increasing pattern after 400 s could be used to detect the conditions of the concrete under the cyclic loading [54]. Both BOF20 and FA20 showed the best repeatability with the mean values of FCR as −6% and −1.5%, respectively. In BOF20, particularly, FCR was increased with the loading, which could be explained by, and thus used to indicate the occurrence of the irreversible cracks. The highest FCR results was observed when red mud was under the large amplitude of loading (18 MPa). Figure 10b shows the patterns of FCR for FA25, BOF25 and RM25. In general, FA25 and RM25 performed comparable patterns comparing with FA20 and RM20, respectively. However, BOF25 was observed with the significantly different pattern during the loading time of 0–300 s. On the other hand, the variation of FCR was not stable when RM was used. In addition, FCR of all the specimens did not return to their initial values, which could be related to the elastic regime of these specimens [55]. FA might be the most suitable to use for the self-sensing concrete.

## 6. Conclusions

In this study, we reported the environment-friendly, self-sensing concrete. The self-sensing concrete was fabricated with the byproduct wastes (i.e., coal fly ash, blast furnace slag and red mud) and cured using the conditions of 3, 7 and 28 days. The self-sensing concrete samples were experimentally tested to investigate the effects of the byproduct wastes on the mechanical and electrical properties (i.e., compressive strength and fractional change of resistivity FCR). In the end, parametric studies were experimentally conducted to investigate the influences of the byproduct wastes on the FCR the reported concrete. The main findings can be drawn as: (1) The addition of byproducts wastes was observed to significantly affect the electrical properties (i.e., FCR) of the reported concrete samples, (2) FCR can be used to indicate the working conditions of the reported concrete for the purpose of self-sensing and (3) FA might be the most suitable addition for the self-sensing concrete since the obtained FCR were more stable and reliable comparing with other additions. 

## Figures and Tables

**Table 1:** Chemical composition (wt.%) and loss on ignition (wt.%) of the materials.

	SiO_2_	CaO	Al_2_O_3_	Fe_2_O_3_	MgO	MnO	K_2_O	Na_2_O	SO_3_	TiO_2_	P_2_O_5_	LOI
Portland cement	19.2	61.7	4.7	2.3	2.6	-	1.03	0.24	3.7	-	-	1.3
Coal fly ash (FA)	*36.24*	*10.99*	*23.7*	9.76	2.75	-	1.72	0.38	3.97	-	-	3.45
Blast furnace slag (BOF)	*15.6*	*44.7*	*3.9*	18.4	7.2	4.4	-	-	0.8	0.4	1.7	2.5
Red mud (RM)	9.6	19.3	20.7	31.8	0.3	-	-	1.9	-	7.3	0.8	6.15

**Table 2:** Specimen description and water/cement ratio.

Additives Type and Fraction (%)	Description	W/C Ratio
Ordinary Portland cement (OPC)	100% Portland cement	0.56
Coal fly ash type F (FA5)	5% fly ash and 95% Portland cement	0.56
FA10	10% fly ash and 90% Portland cement	0.56
FA15	15% fly ash and 85% Portland cement	0.53
FA20	20% fly ash and 80% Portland cement	0.53
FA25	25% fly ash and 75% Portland cement	0.50
Blast furnace slag (BOF5)	5% steel slag and 95% Portland cement	0.53
BOF10	10% steel slag and 90% Portland cement	0.51
BOF15	15% steel slag and 85% Portland cement	0.50
BOF20	20% steel slag and 80% Portland cement	0.50
BOF25	25% steel slag and 75% Portland cement	0.50
Red mud (RM5)	5% red mud and 95% Portland cement	0.53
RM10	10% red mud and 90% Portland cement	0.53
RM15	15% red mud and 85% Portland cement	0.51
RM20	20% red mud and 80% Portland cement	0.51
RM25	25% red mud and 75% Portland cement	0.50

**Table 3:** Setting time and pH results for different mixes.

Specimens	Initial Setting Time (min)	Final Setting Time (min)	pH
OPC	186	480	12.90
FA5	210	496	12.92
FA10	233	509	12.82
FA15	247	512	12.73
FA20	312	515	12.55
FA25	356	523	12.44
BOF5	188	451	12.68
BOF10	195	457	12.87
BOF15	201	459	12.64
BOF20	205	462	12.55
BOF25	217	468	12.52
RM5	173	470	12.92
RM10	168	463	12.76
RM15	165	457	12.70
RM20	157	439	12.55
RM25	150	435	12.53

**Table 4:** Stress sensitivity coefficient results for cement mortars at the age of 28 days.

Specimen	Stress Sensitivity Coefficient (MPa^−1^) × 10^3^
OPC	4.34
FA5	6.25
FA10	7.17
FA15	0.85
FA20	8.41
FA25	3.13
BOF5	1.26
BOF10	5.44
BOF15	1.25
BOF20	5.35
BOF25	1.09
RM5	3.27
RM10	4.18
RM15	1.34
RM20	2.26
RM25	6.29

## Appendix A

1. Raw data of the compressive strength and electrical resistance for the environment-friendly, self-sensing concrete. To avoid measurement errors existed in the experiments, we repeated the same experiments three times and the presented raw testing data are the mean values.
2. Table A1 presents the results for the concrete samples cured at the age of 3 days, which leads to Figure 6 in the main text. Table A2 presents the results for the concrete samples cured at the age of 7 days, which leads to Figure 7 in the main text. Table A3 presents the results for the concrete samples cured at the age of 28 days, which leads to Figure 8 in the main text.

**Table A1:** Compressive strength and electrical resistance results for the environment-friendly, self-sensing concrete cured at the age of 3 days.

Specimen	Compressive Strength (MPa)	Electrical Resistivity (mΩ)
OPC	16.65	10.60
FA5	13.93	9.20
FA10	14.29	8.10
FA15	14.68	5.80
FA20	15.09	5.20
FA25	15.19	3.50
BOF5	16.64	7.50
BOF10	15.73	5.10
BOF15	13.44	4.30
BOF20	12.89	3.50
BOF25	11.74	2.10
RM5	14.29	8.75
RM10	14.85	7.60
RM15	14.22	6.85
RM20	12.96	5.65
RM25	12.46	3.20

**Table A2:** Compressive strength and electrical resistance results for the environment-friendly, self-sensing concrete cured at the age of 7 days.

Specimen	Compressive Strength (MPa)	Electrical Resistivity (mΩ)
OPC	22.57	35.10
FA5	20.93	41.30
FA10	20.75	47.10
FA15	20.02	39.70
FA20	18.28	31.60
FA25	17.44	30.20
BOF5	21.49	28.60
BOF10	21.21	25.10
BOF15	20.24	21.40
BOF20	19.11	19.45
BOF25	17.43	17.90
RM5	18.32	29.20
RM10	17.23	27.10
RM15	16.95	23.60
RM20	15.94	21.90
RM25	15.82	18.60

**Table A3:** Compressive strength and electrical resistance results for the environment-friendly, self-sensing concrete cured at the age of 28 days.

Specimen	Compressive Strength (MPa)	Electrical Resistivity (mΩ)
OPC	32.65	113.20
FA5	27.94	88.50
FA10	26.46	79.30
FA15	24.57	58.10
FA20	23.08	64.30
FA25	22.17	53.10
BOF5	32.58	37.60
BOF10	29.27	36.70
BOF15	28.25	33.25
BOF20	26.12	31.60
BOF25	24.45	28.25
RM5	31.27	39.70
RM10	32.94	39.10
RM15	27.52	38.60
RM20	25.83	37.80
RM25	25.15	36.50
